# Algae and Their Metabolites as Potential Bio-Pesticides

**DOI:** 10.3390/microorganisms10020307

**Published:** 2022-01-27

**Authors:** Elias Asimakis, Awad A. Shehata, Wolfgang Eisenreich, Fatma Acheuk, Salma Lasram, Shereen Basiouni, Mevlüt Emekci, Spyridon Ntougias, Gökçe Taner, Helen May-Simera, Mete Yilmaz, George Tsiamis

**Affiliations:** 1 Laboratory of Systems Microbiology and Applied Genomics, Department of Environmental Engineering, University of Patras, 2 Seferi St., 30131 Agrinio, Greece; eliasasim@gmail.com; 2 Research and Development Section, PerNaturam GmbH, 56290 Gödenroth, Germany; awad.shehata@pernaturam.de; 3 Bavarian NMR Center—Structural Membrane Biochemistry, Department of Chemistry, Technische Universität München, 85748 Garching, Germany; wolfgang.eisenreich@mytum.de; 4 Laboratory for Valorization and Conservation of Biological Resources, Faculty of Sciences, University M’Hamed Bougara of Boumerdes, Boumerdes 35000, Algeria; fatma.acheuk@yahoo.fr; 5 Laboratory of Molecular Physiology of Plants, Borj-Cedria Biotechnology Center. BP. 901, Hammam-Lif 2050, Tunisia; Salma.lasram.cbbc@gmail.com; 6 Institute of Molecular Physiology, Johannes Gutenberg-University of Mainz, 55128 Mainz, Germany; shereen.basiouni@klinik.uni-regensburg.de (S.B.); hmaysime@uni-mainz.de (H.M.-S.); 7 Department of Plant Protection, Faculty of Agriculture, Ankara University, Keçiören, Ankara 06135, Turkey; Mevlut.Emekci@agri.ankara.edu.tr; 8 Department of Environmental Engineering, Democritus University of Thrace, Vas. Sofias 12, 67132 Xanthi, Greece; sntougia@env.duth.gr; 9 Department of Bioengineering, Bursa Technical University, Bursa 16310, Turkey; gokce.taner@btu.edu.tr

**Keywords:** cyanobacteria, algal extracts, antimicrobial, plant defense, photosynthesis

## Abstract

An increasing human population necessitates more food production, yet current techniques in agriculture, such as chemical pesticide use, have negative impacts on the ecosystems and strong public opposition. Alternatives to synthetic pesticides should be safe for humans, the environment, and be sustainable. Extremely diverse ecological niches and millions of years of competition have shaped the genomes of algae to produce a myriad of substances that may serve humans in various biotechnological areas. Among the thousands of described algal species, only a small number have been investigated for valuable metabolites, yet these revealed the potential of algal metabolites as bio-pesticides. This review focuses on macroalgae and microalgae (including cyanobacteria) and their extracts or purified compounds, that have proven to be effective antibacterial, antiviral, antifungal, nematocides, insecticides, herbicides, and plant growth stimulants. Moreover, the mechanisms of action of the majority of these metabolites against plant pests are thoroughly discussed. The available information demonstrated herbicidal activities via inhibition of photosynthesis, antimicrobial activities via induction of plant defense responses, inhibition of quorum sensing and blocking virus entry, and insecticidal activities via neurotoxicity. The discovery of antimetabolites also seems to hold great potential as one recent example showed antimicrobial and herbicidal properties. Algae, especially microalgae, represent a vast untapped resource for discovering novel and safe biopesticide compounds.

## 1. Introduction

The term algae encompasses a wide range of photosynthetic organisms that are found primarily in freshwater and marine environments, although certain representatives can thrive in terrestrial niches, either on their own or by developing symbiotic relationships with other organisms [1,2]. Larger eukaryotic algae, such as seaweeds, are called macroalgae, while smaller unicellular eukaryotes are collectively referred to as microalgae. On the other hand, cyanobacteria (blue-green algae) represent the prokaryotic clade of this highly diverse group [2]. Moreover, some genera exhibit extraordinary growth potential in terms of produced biomass within a short period of time [3,4].

Due to the high biomass production potential and their ability to grow relatively quickly, recent research on algae has been primarily focused on biofuel production. They are more promising alternatives than plant-based biofuel production, because, unlike plants, they do not require fertile land for growth; thus, they do not interfere with food production [2,5,6]. However, significant research effort has been made in recent years in identifying and isolating bioactive compounds from algae. These compounds may possess pharmaceutical or biomedical value and can be used in anticoagulant, antioxidant, antitumor, antimicrobial, immunomodulatory, antilipidemic, hypoglycemic, and anti-inflammatory products [7,8,9,10]. Algae and their metabolites can also be used in cosmetics due to their antioxidant or tissue regenerative action and in the food industry, due to their high content of fibers, minerals, vitamins, pigments, and antioxidants [8]. In terms of biochemical properties, these metabolites can be lipids, proteins, peptides, polysaccharides, carotenoids, phenolics, and alkaloids [5,11]. Such bioactive compounds can be produced on an industrial and agricultural scale, providing substantial economic benefits [8]. Furthermore, recent advancements in microalgae biotechnology have made it possible to use microalgae as bioreactors for recovering recombinant proteins and medicinal products such as vaccines, antibodies, immunotoxins, and antimicrobial agents [3,12].

Algae and their extracts also display antimicrobial, nematocidal, herbicidal, and insecticidal/acaricidal properties against crop pathogens and can be used as biopesticides [13,14,15]. The term biopesticide may encompass naturally occurring compounds (e.g., sodium bicarbonate, sodium acetate), biochemical substances that are produced naturally by various organisms or by genetically-modified plants, as well as microorganisms (bacteria, viruses, fungi, algae etc.) that are used to control pests in agricultural practices. In this regard, algal components have been shown to efficiently suppress plant pathogenic bacteria, including the genera *Agrobacterium*, *Pseudomonas*, *Xanthomonas*, and *Erwinia*, which are associated with serious diseases of important crops that form the nutritional basis of most human societies, such as rice and potato plants [16,17,18]. Similar effects have been identified against the most common plant pathogens, fungi. Algal extracts can inhibit mycelial growth and induce resistance in plants against widespread fungal genera such as *Fusarium*, *Verticillium*, *Rhizoctonia*, *Phytophthora*, and *Phoma* [18,19,20]. Their antimicrobial activity also expands to viral pathogens, such as tobacco mosaic virus (TMV) and potato virus X (PVX), which are inhibited either directly or by inducing plant defense mechanisms [21,22,23]. As well as effectively controlling pathogenic microorganisms, algal extracts have been tested with success against animal targets. These targets include soil-borne nematodes, plant or fruit feeding insects (e.g., fruit fly larvae) and mites, as well as insects that mediate transmission of diseases [24,25,26,27]. Herbaceous weeds that hinder crop development, as well as algal species that grow uncontrollably resulting in harmful algal blooms, can also be treated with algal products. The effect in these cases, are either based on cytotoxicity or the inhibition of photosynthesis [28,29].

The use of natural products against pests in agricultural practices instead of synthetic chemicals shows significant advantages. These compounds are environmentally safe due to their high biodegradability and low residuality, are safer to non-target organisms due to better specificity, and are less likely to produce resistance due to diverse mechanisms of action [30]. Besides, there is an increasing interest to identify and recover such bioactive compounds from various kinds of algae that can be used for plant protection purposes in the context of sustainable agricultural practices. This review focuses on the current knowledge of algal bioactive substances with known pesticidal action.

## 2. Biological Roles of Algal Compounds or Extracts

### 2.1. Antibacterial Action

An array of diverse chemical compounds from algae, including alkaloids, polyketides, peptides, polysaccharides, phlorotannins, diterpenes, sterols, quinones, lipids, and glycerols, have been found to exhibit antibacterial action [14,31]. However, in some instances, the specific compounds with antibacterial properties are not fully elucidated, and the activity is collectively attributed to algal extracts. Also, as most studies focus on the antibacterial properties of algal extracts on human pathogens, information on plant pathogens is scarce but is constantly being enriched. An excellent example of algal extracts with antibacterial action comes from the brown marine alga *Sargassum wightii* (Table 1). 

**Figure 1:**
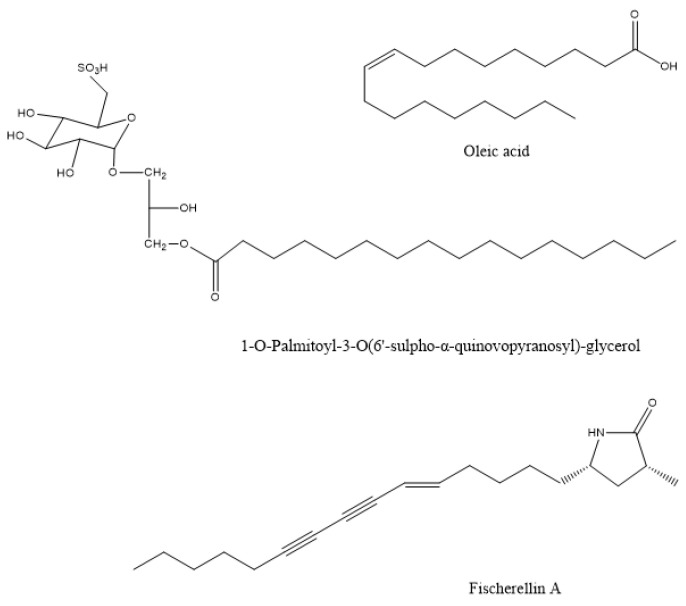
The chemical structure of active compounds that were isolated from *Sargassum wightii*, *Padina gymnospora*, and *Fischerella muscicola*.

The methanolic extracts of this species showed maximum antibacterial activity against the plant pathogen *Pseudomonas syringae*, to prevent leaf spot disease of the medicinal herb *Gymnema sylvestre* [32]. Similar in vitro activity against the phytopathogenic bacterium *Xanthomonas oryzae* was observed by the macroalgae *Gracilaria edulis*, *Sargassum wightii*, and *Enteromorpha flexuosa* [16]. The compound that was responsible for this action in the brown alga *Sargassum wightii* was the sulphoglycerolipid 1-O-palmitoyl-3-O(6′-sulpho-a-quinovopyranosyl)-glycerol (Figure 1) from the methanolic extract [33]. The plant pathogens *Xanthomonas campestris* and *Erwinia carotovora* were also inhibited in vitro by the methanol-soluble and insoluble extracts of *Ulva fasciata* [34]. The spray application of aqueous extracts from the brown algae *Cystoseira myriophylloides* and *Fucus spiralis* significantly reduced Crown gall disease incidence that was caused by the bacterial pathogen *Agrobacterium tumefaciens* in greenhouse tomato plants (*Solanum lycopersicum*) [18]. The response was related to oxidative burst mechanisms since the treated plants exhibited significantly greater activity levels of the plant defense enzymes polyphenol oxidase and peroxidase. The methanolic extract of the marine brown algae *Sargassum latifolium*, *Hydroclathrus clathratus*, and *Padina gymnospora* showed antibacterial activity against the soil-borne phytopathogenic bacteria *Ralstonia solanacearum* and *Pectobacterium carotovorum*. The extract of *Padina gymnospora,* which showed the most potent effect, was dominated by palmitic and oleic acids (Figure 1) [35].

**Figure 2:**
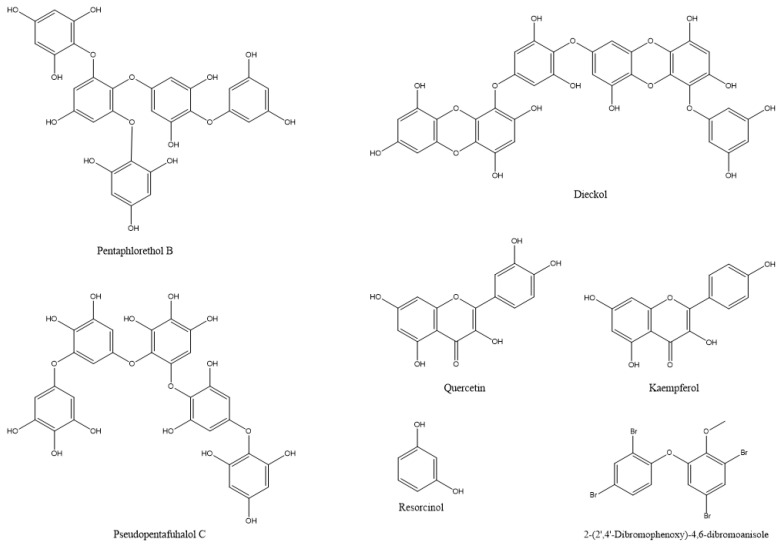
The chemical structures of active compounds that were isolated from *Cladaphora fascicularis* and *Arthrospira platensis* and other microalgae.

**Figure 3:**
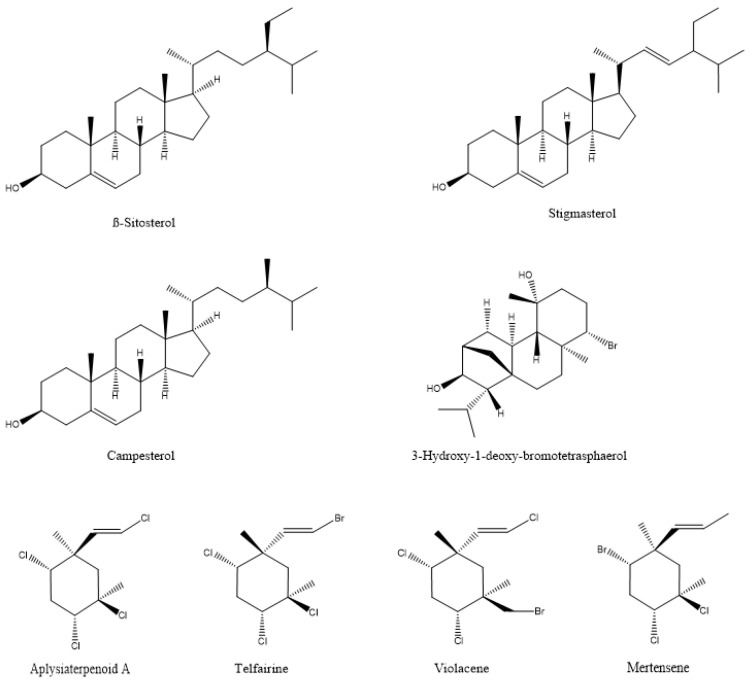
The chemical structure of active compounds that were isolated from *Sphaerococcus coronopifolius*, *Plocamium cartilagineum*, *Plocamium telfairiae*, and *Prasiola crispa*.

In most documented cases, the antibacterial activity is against human or animal pathogens. In terms of agricultural practices, this is important for pathogenic bacteria that are associated with livestock. However, such agents could be further tested as potential candidates for plant protection applications, leaving ample room for future research [19,36,37,38,39,40,41,42,43,44,45,46,47]; such are the cases of the tetrabrominated diphenyl ether-like 2-(2′,4′-dibromophenoxy)-4,6-dibromoanisole (Figure 2) that was isolated from the green alga *Cladaphora fascicularis* that showed bactericidal action against *Escherichia coli*, *Bacillus subtilis*, and *Staphylococcus aureus* [38], and tetracyclic brominated diterpenes such as 3-hydroxy-1-deoxy-bromotetraspaerol (Figure 3) from the red alga *Sphaerococcus coronopifolius* that exhibited antibacterial activity against a panel of *S. aureus* strains [36]. Interestingly, ethyl acetate extracts from two cyanobacterial species, *Anabaena variabilis* and *A*. *circinalis*, were effective against bacterial fish pathogens belonging to the genus *Aeromonas*, proving to be useful in aquaculture applications [48]. Moreover, as most of the published studies focus on macroalgae or cyanobacteria’s antimicrobial activity, microalgae could constitute another exciting prospect for future research.

### 2.2. Antiviral Action

**Figure 4:**
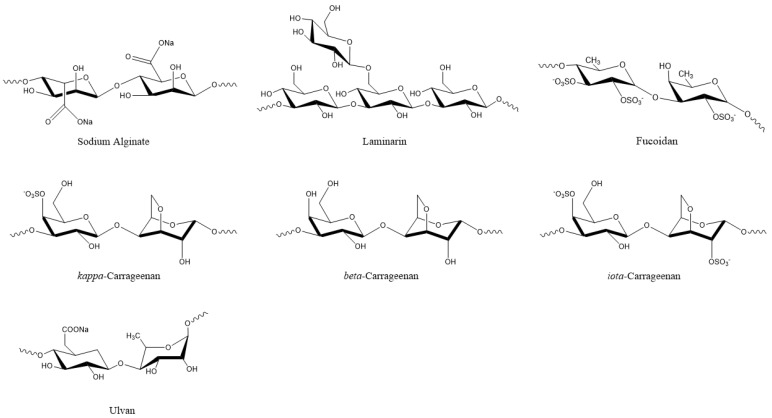
Algal polysaccharides with pesticidal action.

Plant viruses are a serious threat to agricultural crops, affecting product quality and yields and resulting in severe economic losses [30]. Their management is heavily dependent on synthetic chemical products, but natural compounds are continuously gaining ground. In this respect, natural compounds from algae that exhibit antiviral properties could be valuable resources. Among them are various polysaccharides, such as laminarins, agarans, alginate, carrageenans, and sulphated fucans, that can function as elicitors of defense mechanisms, as well as proteins, lipids, tannins, and terpenoids [10,13,30,50,51]. Polysaccharides are the most common compounds in algal extracts that induces antiviral responses in plants. For example, sodium alginate (Figure 4) from marine algae exhibited strong inhibitory activity against the tobacco mosaic virus (TMV) that was isolated from systemically-infected leaves of *Nicotiana tabacum* L. var bright yellow [21] (Table 2). 

The degree of inhibition increased with alginate concentration and was higher when the alginate polymer had a lower mannuronate to guluronate ratio [21]. Another polysaccharide, i.e., kappa/beta-carrageenan (Figure 4) from red marine alga *Tichocarpus crinitus*, reduced tobacco mosaic virus (TMV) infection in Xanthi-nc tobacco leaves by 87 percent [52]. The same compound stimulated lytic processes against PVX particles in the leaves of *Datura stramonium* [22]. Methanolic extracts from six algal species also showed inhibitory effects against PVX by more than 80%. Among them, the extract from *Fucus gardneri* contained the polysaccharide alginate, which showed 95% success in suppressing PVX infection via aggregation of the virus particles [23].

Apart from polysaccharides, lectins (carbohydrate-binding proteins) that were isolated from the marine alga *Ulva pertusa* also showed antiviral action against tobacco mosaic virus (TMV) [53,54]. Similarly, lipids that were extracted from 11 algal species displayed antiviral activity against TMV on *Nicotiana tabacum* cv. Xanthi-nc. The brown alga *Cystoseira balearica* and the red alga *Lophocladia lallemandii* showed the highest inhibitory activity [19]. Aqueous and ethanolic extracts from the brown alga *Durvillaea antarctica* suppressed damage that was caused by TMV in tobacco leaves [17]. Moreover, an exciting application in livestock animals refers to the use of sulphated polysaccharides from the algae *Ulva clathrata* and *Cladosiphon okamuranus* in inhibiting Newcastle Disease Virus (NDV) infection in poultry [55].

Research for algal compounds with antiviral effects in plants has yielded significant results in the past years and remains promising for discovering new biopesticide products. However, as expected, algal antiviral research is mainly concentrated around human pathogens. Algal extracts have been studied extensively for their inhibitory action against important human viruses, such as human immunodeficiency virus (HIV), human papilloma virus (HPV), hepatitis B virus (HBV), herpes simplex virus types 1 and 2 (HSV-1, HSV-2), and diverse strains of Dengue virus (DENV-2) [56,57,58,59,60,61,62,63,64], suggesting that there are still many research opportunities in the field of algal antiviral products.

### 2.3. Antifungal Action

Natural algal compounds are constantly gaining ground in modern agricultural practices in controlling fungal infection, one of the most common types of disease in cultivated plants. They are mostly preferred over synthetic products, due to lower environmental impact, high specificity, and performance [14,51]. Algal powders and a large variety of extracts, such as aqueous, methanolic, ethanolic, diethyl ether, acetone, ethyl acetate, benzene, and chloroform, have proven to be effective in protecting plants against pathogenic fungal species [17,18,20,24,35,65,66,67,68,69,70,71,72,73,74,75,76,77]. For instance, ethanolic extracts of the cyanobacterium *Nostoc* strain ATCC 53,789 inhibited the growth of nine fungal plant pathogens (Table 3). 

The extract, which was directly applied on tomato plants, completely inhibited the growth of the fungus *Sclerotinia sclerotiorum* [20]. The aqueous extracts of three brown algae *Cystoseira myriophylloides*, *Laminaria digitata*, and *Fucus spiralis*, that were applied as spray or drench, had a protective effect against Verticillium wilt disease that was caused by the fungus *Verticillium dahliae*, in greenhouse tomato seedlings [18]. In tomato leaves that were infected with *Botrytis cinerea*, organic extracts from the brown alga *Lessonia innamomic* reduced the frequency and extent of necrotic lesions, while aqueous and ethanolic extracts from the red alga *Gracillaria chilensis* were effective against *Phytophthora innamomic*, showing dose and time-dependent responses [17]. Methanolic extracts of the sea brown algae *Sargassum latifolium* and *Padina gymnospora* showed antifungal activity against phytopathogenic fungus species such as *Fusarium solani* and *Rhizoctonia solani* in addition to antibacterial activity [35]. Notably, the antifungal activity of alkaline extracts of *Ulva lactuca*, *Sargassum filipendula*, and *Gelidium serrulatum* has been linked to the increase in plant defense enzymes activity and the overexpression of key marker genes of plant defense pathways [49].

De Corato et al. [68] performed in vitro and in vivo tests of extracts from two brown and three red macroalgae against three phytopathogenic fungi. The compounds that were identified in the extracts included twenty fatty acids, three types of polysaccharides (laminarans, fucoidans, and alginates) (Figure 4), and three types of phlorotannins (phlorethols, fucophloretols, and eckols) (Figure 2). Regarding lipids, palmitic acid, linoleic acid, and arachidonic acid were found at the highest concentrations in most extracts. The quantity of fatty acids corresponded to most of the total dry weight of the crude extracts and could be linked to the antifungal activity [68]. Lipids that were extracted from 10 algal species inhibited the germination of *Phoma tracheiphila*, a pathogenic fungus that causes a disease known as Mal secco on citrus trees. More specifically, the inhibition effect of the brown alga *Cystoseira balearica* and the green alga *Codium effusum* reached 100% [19].

Various algal oligo- or polysaccharides have demonstrated antifungal activity against plant pathogens either directly or indirectly, by activating plant defense mechanisms [78,79,80,81,82,83,84]. The cell wall polysaccharide laminarin (Figure 4) that was purified from the brown algae *Laminaria digitata* induced a defense response in grapevine leaves against the fungus *Botrytis cinerea*, effectively reducing the infection. In addition, grapevine plants that were sprayed on the leaves with laminarin were protected against the fungus *Plasmopara viticola* [85]. Polysaccharides from *Anabaena* sp., *Ecklonia* sp., and *Jania* sp. showed inhibitory action against *Botrytis cinerea*, effectively protecting strawberry fruits from infection in in vitro experiments [86]. Similar polysaccharide-rich extracts from green (*Ulva lactuca* and *Caulerpa sertularioides*) and brown (*Padina gymnospora* and *Sargassum liebmannii*) macroalgae induced resistance in tomato plants against the necrotrophic fungus *Alternaria solani* [87]. The water-soluble heteropolysaccharide ulvan (Figure 4) from *Ulva* sp. extracts significantly reduced the severity of *Glomerella* leaf spot (GLS) disease, caused by the fungus *Colletotrichum gloeosporioides* on the leaves of apple plant seedlings (*Malus domestica*). The induced resistance was associated with increased peroxidase activity, revealing that although ulvan does not exhibit antimicrobial activity, its action is associated with plant defense mechanisms [88].

Extracts and powders of the green seaweeds *Ulva fasciata* and *Enteromorpha flexuosa* inhibited the growth or affected the microsclerotia formation of the soil-borne fungi, *Macrophomina phaseolina* and *Fusarium solani*, that infect cucumber plants. The identification of iron-monocarbonyl and their functional groups, such as amine and ether in the extracts, could suggest a potential antifungal role for these compounds [89]. Similar compounds were also identified in the chloroform extracts from the red macroalgae *Gracilaria confervoides* that exhibited inhibitory action against three soil-borne pathogenic fungi of cucumber: *Rhizoctonia solani*, *Fusarium solani*, and *Macrophomina phaseolina* [75].

The methanolic extract of the brown alga *Sargassum vulgare* contained phenolic acids and flavonoids that may be responsible for the antifungal action that was observed against *Pythium aphanidermatum*, the causative agent of Pythium leak disease in potato [90]. Phenolic acids and phytoalexins were among the 18 compounds that were detected in the acetone extract of the brown alga *Sargassum wightii*. The extract exhibited antifungal activity against *Rhizoctonia solani*, the causative agent of rice sheath blight [91].

### 2.4. Nematocidal Action

The extracts and compounds from micro- and macroalgae are also effective against plant-parasitic nematodes that are responsible for the annual loss of 10–25% of worldwide crop production [92] (Table 4). 

Brominated diterpenes that were isolated from the marine red alga *Jania rubens* were effective against *Allolobophora caliginosa* [93]. Different concentrations of methanolic extracts from the *Nostoc* strain ATCC 53,789 either killed or slowed the development of the nematode *C. elegans* [20]. Dry powders from three macroalgae species, *Spatoglossum variabile*, *Stokeyia indica*, and *Melanothamnus afaqhusainii* displayed a suppressive effect on the root-knot nematode *Meloidogyne incognita* by reducing the gall formation and preventing nematode penetration in the roots of eggplant and watermelon [65]. Similar effects against the nematode *Meloidogyne javanica* were identified in okra, sunflower, and tomato after treatment with aqueous and ethanolic extracts of the marine macroalgae *Sargassum tenerrimum*, *S. swartzii*, *S. wightii*, *Spatoglossum variabile*, *Melanothamnus afaqhusainii*, and *Halimeda tuna* [24,94]. Algal extracts showed relatively similar suppressive effects with a carbofuran nematicide; however, the best result was obtained when extracts from *Spatoglossum variabile* were applied together with the synthetic product [24]. The marine alga *Stoechospermum polypodioides* was also effective against *Meloidogyne javanica*, causing 80% mortality, the strongest effect among 21 species of algae that were examined for their nematocidal action [95]. A reduction in root-knot nematode infestation using macroalgal extracts has been previously reported in tomato [96]. There are two commercially available products that are derived from the marine macroalgae *Ascophyllum nodosum* and *Ecklonia maxima*, that affected the hatching and sensory perception of the root-knot nematodes *Meloidogyne chitwoodi* and *M. hapla*. The alkaline extract from the brown marine alga *A. nodosum* showed a stronger inhibitory effect compared to the extract from the brown alga *E. maxima*, which in certain cases enhanced the infectivity of the nematodes [97]. Extracts of *A. nodosum* have shown inhibitory action against other nematodes, such as *Radopholus similis*, *Meloidogyne incognita*, and *Belonolaimus longicaudatus*, infecting citrus, tomato, and centipede grass, respectively [98,99,100,101].

### 2.5. Insecticidal—Acaricidal Action of Algae

Marine macroalgae extracts exhibit insecticidal/acaricidal activity and can be used in integrated pest management applications as environmentally friendly approaches for arthropod population control. These botanical biopesticides are safer than synthetic products and are equally or more effective since they display different modes of action. Therefore, their targets are less likely to develop resistance against them [102,103]. There are a large number of publications describing the pesticidal or repellent action of algal extracts against different arthropods that are either agricultural pests or related to human and animal health [25,26,104,105,106,107,108,109,110,111,112,113,114,115]. The bioactive compounds that were identified in the extracts, as expected, cover a broad range of chemical structures, including polysaccharides, phenolics, proteins, terpenes, lipids, and halogenated compounds [116]. For example, two halogenated monoterpenes (mertensene and violacene) (Figure 3) that were extracted from the red alga *Plocamium cartilagineum*, and the mertensene derivatives, dibromomertensene, and dihydromertensene, showed strong insecticidal activity against the tomato moth *Tuta absoluta* and the cereal aphid, *Schizaphis graminum* [104] (Table 5). 

Water and ethanol extracts from the cyanobacterium *Arthrospira platensis* (syn. *Spirulina platensis*) and the brown alga *Sargassum vulgar* contained phenols, tannins, and alkaloids. Further analysis of phenols in *A. platensis* revealed the presence of the phenolic compounds: quercetin, kaempferol, and resorcinol (Figure 2), which could be related to the insecticidal action of the extracts [27]. An evaluation of the ethanolic extracts of the macroalgal species (*Caulerpa sertularioides*, *Laurencia johnstonii*, and *Sargassum horridum*) revealed insecticidal and repellent activities against Asian citrus psyllid adults (*Diaphorina citri*). The chemical composition of the three extracts showed phenols, alkaloids, terpenes, tannins, flavonoids, saponins, and anthraquinones, which are associated with insecticidal and repellent activity [109]. In certain cases, algal extracts exhibit different insecticidal effects, depending on the insect species and on the developmental stages of the target insect. For instance, the acetone extract of *Ulva lactuca* was the most potent extract against *Culex pipiens*. In the case of the cotton leafworm, *Spodoptera littoralis*, ethanolic and chloroform extracts acted as larvicides, methanolic and ethanolic extracts resulted in the highest pupation inhibition, whereas etheric and methanolic extracts strongly inhibited larval growth and adult emergence [117]. In terms of agricultural pests, more research has focused on the insecticidal activity of algal extracts against the cotton insect pests of the genera *Dysdercus* and *Spodoptera*. Algal extracts of various species, including *Caulerpa scalpelliformis* [118], *Padina pavonica* [115], *Sargassum tenerrimum* [114], *Sargassum vulgar* [27], *Ulva fasciata*, and *U. lactuca* [105] were effective against these agricultural pests by causing nymphal mortality, adult mortality, abnormal development, or reducing adult lifespan, fecundity, and hatchability [14,106]. As observed throughout the text, algal extracts could be used against more than one target type (antimicrobial, insect, mite, etc.). Therefore, apart from the antifungal action, the methanolic extracts of the cyanobacterium *Nostoc* strain ATCC 53,789 also killed larvae of the moth *Helicoverpa armigera* [20].

The volatile oils from *Actinotrichia fragilis*, *Liagora ceranoides*, and *Colpomenia sinuosa* were hydrodistilled and contained different aliphatic alcohols and long-chain hydrocarbons as the major components. They caused 55–90% and 60–80% mortality to pests of stored products *Oryzaephilus mercator* and *Tribolium castaneum*, respectively, at a dose of 12 μL/L air after 48 h of fumigation exposure [119].

The volatile oils of *A. fragilis* caused 80–90% mortality in both *T. castaneum* and *O. mercator* and consisted of 49% aliphatic alcohols, mainly 1-dodecanol (39.6%) [119]. The insecticidal activity of 1-dodecanol was suggested to be related to the developing cuticle, producing a disruption in the cuticular tanning process [120].

### 2.6. Herbicidal Activity

**Figure 5:**
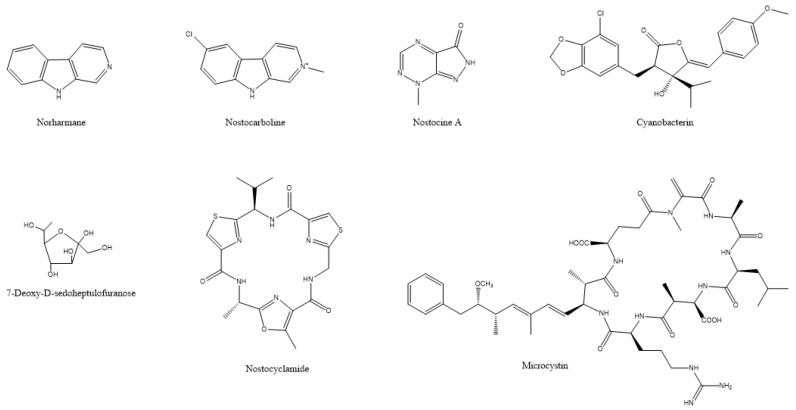
The chemical structures of active compounds that were isolated from *Synechocystis aquatilis*, *Scytonema hofmanni*, *Nostoc* sp., *Microcystis aeruginosa*, and *Synechococcus elongatus*.

Natural bioherbicides have become a useful tool for the integrated management of herbaceous weeds, a major problem in agriculture that are known for impairing the growth of cultivated plants and effectively reducing crop yields [121]. Various algal metabolites, such as cyanotoxins that exhibit cytotoxicity, have been considered as potential candidates for herbicidal activity [6]. In many documented cases, cyanotoxins have ecological role as allelochemicals, inhibiting competitive macrophytes, algae, and microbes [122,123,124,125]. Such is the case in the recovery of indole alkaloid norharmane (Figure 5) from the cyanobacterium *Synechocystis aquatilis*, which inhibited the growth of the cyanobacteria *Microcystis aeruginosa* and *Oscillatoria limnetica*, and that of the green algae *Chlorella vulgaris* and *Ulothrix* sp. [126] (Table 6). 

Although this action was not against land crop weeds but algal blooms, it has been found that norharmane can also inhibit the germination of weeds, such as *Avena fatua* and *Plantago lanceolata*; suppress the growth of *Portulaca oleracea*, *Echinochloa crus-galli* and *Amaranthus retroflexus*; and damage the metabolism in *Arabidopsis thaliana* [28]. Cyanobacterin (a chlorinated γ-lactone) (Figure 5) is another allelopathic substance that was isolated from the freshwater cyanobacterium *Scytonema hofmanni* that is highly toxic to other cyanobacteria and eukaryotic algae by interrupting photosynthetic electron transport [29]. There are two other structurally different cyanobacterins that were identified in *Nostoc linckia* that specifically inhibit electron transport in photosystem II [127,128]. A series of metabolites, such as nostocyclamide, nostocine A and nostocarboline (Figure 5) that were isolated from a *Nostoc* sp., exhibited toxic action against algae [129,130,131]. Another type of toxin, microcystins (for an example see Figure 5) that were isolated from *Microcystis aeruginosa,* inhibits the growth of aquatic plants, such as *Myriophyllum variifolium* and *Lemna japonica* [132,133]. A sugar, 7-deoxy-sedoheptulose (7dSh) (Figure 5) that was isolated from the cyanobacterium *Synechococcus elongatus*, inhibited the growth of various phototrophic organisms, including cyanobacteria as well as yeasts and plants [134]. Moreover, the methanolic extract of the *Nostoc* strain ATCC 53,789 hindered the development of a mixture of grass seedlings by reducing culm height and inhibiting root development, even though it did not affect the initial germination of the seeds [20]. However useful, one of the limitations of cyanobacterial toxins is their potential toxic effects against non-target organisms, including humans, animals, and plants, and their bioaccumulation in the food chain. Therefore, designing candidate agricultural products that make use of toxins should always take into consideration the adverse effects to non-target organisms [122,124].

### 2.7. Plant Growth Stimulation (Biostimulators and Biofertilizers) and Bioprotection

Algal extracts and compounds are valuable resources that display a wide range of beneficial effects on cultivated plants. Such effects include improved seed germination, plant growth, crop performance and yield, post-harvest shelf-life, and protection against biotic and abiotic factors [135,136,137,138,139]. Compounds with growth-stimulating activities include polysaccharides, minerals, trace elements, growth hormones, betaines, and sterols [137].

Alkaline extracts of *Ascophyllum nodosum* enhanced the development of the mycorrhizal fungus *Rhizophagus irregularis* in terms of spore germination, germ tube length, and hyphal branching. The application of the extracts improved the formation of mycorrhizal associations, increased root colonization, arbuscular maturity, and the size of greenhouse-grown *Medicago truncatula* plants [140]. An *Ascophyllum nodosum* extract significantly improved the root, shoot, and fruit biomass productivity parameters by positively influencing the rhizospheric bacterial and fungal communities that are associated with pepper and tomato plants [141]. Additionally, beneficial effects of an alkaline extract of *A. nodosum* were observed in tomato and sweet pepper plants. The foliar application of the extract significantly improved the plant height, leaf number, root length, dry biomass, and increased the leaf chlorophyll content under greenhouse conditions. The extract also reduced disease severity that was caused by pathogenic fungal and bacterial species by 50% under field conditions [142]. The effect of the extract was associated with the induction of plant defense-related enzymes, the production of phenolic compounds, and the activation of genes that were related to auxin, gibberellin, and cytokinin biosynthesis [142]. Plant growth regulators, such as auxin, gibberellic acid, cytokinin, and cytokinin-like compounds, have been isolated from various algal species, including *A. nodosum*, *Ecklonia maxima*, and *Fucus serratus* [143,144,145,146]. The application of *A. nodosum* extract on turf and forage grasses resulted in increased amounts of antioxidant compounds and activity of antioxidant enzymes, therefore, helping plants overcome oxidative stress [147].

Various *Chlorella* species have exhibited similar beneficial effects on the development of cultivated plants. *Chlorella* extracts showed a growth-promoting effect in a variety of crops, including onion, lettuce, cabbage, turnip, wheat, and maize [148,149,150]. A similar effect was also observed in sugar beet with the application of extracts from *Chlorella vulgaris* and *Scenedesmus quadricauda* [151]. Lettuce, tomato, and cucumber seeds that were treated with extracts of *Chlorella vulgaris* were characterized by enhanced germination, higher shoot, and root weights [152,153]. A combination of algal species or compounds could result in more effective microalgae-based fertilizers. For example, powders from *Nannochloropsis* biomass and filamentous microalgae (*Ulothrix* and *Klebsormidium* spp.) effectively enhanced tomato plant growth and improved the fruit quality [154].

Apart from eukaryotic algae, cyanobacteria have also been identified as effective plant biofertilizers. In this respect, composts that were amended with *Anabaena variabilis* and *A. laxa* resulted in enhanced soil organic carbon, nitrogen fixation, significant improvement in growth, yield, fruit quality parameters, nitrogen, phosphorus, and zinc content [73]. *Anabaena variabilis* and a *Nostoc* sp. increased seed germination, plant height, leaf length, and grain yield, when they were applied as biofertilizers on rice seeds or plants [155,156]. Treatment with *Anabaena laxa* resulted in 25% enhanced germination in cumin, while *Calothrix elenkinii* enhanced the root and shoot length significantly in cumin, fennel, and coriander [157]. Filtrates of *Calothrix ghosei*, a *Nostoc* sp. and *Hapalosiphon intricatus*, enhanced the germination, radicle, and coleoptile length in experiments with wheat seeds. Analysis of the filtrates revealed the presence of several amino acids, such as histidine, and auxin-like compounds [158]. The foliar application of seaweed extracts of *Macrocystis pyrifera*, *Bryothamnion triquetrum*, *Ascophyllum nodosum*, *Grammatophora* spp., and *Macrocystis integrifolia* improved the quality of cucumber fruits by increasing the antioxidant capacity of the plant [159]. Moreover, supplementation with *Arthrospira platensis* (*Spirulina platensis*) biomass enhanced the plant growth and improved the seed germination of leafy vegetables, including *Eruca sativa*, *Ameranthus gangeticus*, *Brassica oleracea*, and *Brassica rapa* [160].

The utilization of algal substances could also provide significant benefits to crops in overcoming various types of abiotic stress. Extracts of the brown macroalga *Ascophyllum nodosum* significantly increased the tolerance of *Arabidopsis thaliana* to freezing temperatures, both in vitro and in vivo. The effect was also characterized by reduced tissue, membrane, and chlorophyll damage. Tolerance to freezing stress was also observed when plants were treated only with the lipophilic fraction of the extracts, suggesting that the effect could be associated with its components [161]. Moreover, the extract of *Ascophyllum nodosum* improved the fresh and dry weight of shoots and roots of *Zea mays* that were grown in phosphorous-limited conditions [162]. *Vitis vinifera* plants that were treated with algal extracts exhibited high tolerance against water stress. The extract significantly enhanced potassium and calcium fluxes, improved plant growth, and the accumulated macronutrients in the plant organs [163].

Similarly, *Chlorella vulgaris* induced tolerance against water stress in *Vicia faba* plants [164]. Extracellular products of the cyanobacterium *Scytonema hofmanni* counteracted the effects of salt stress in rice plants. Contrary to salt-stressed plants, treated plants had a higher shoot weight and length, carotenoid content, root weight, and total free porphyrin [165]. A similar effect was induced by *Dunaliella salina* exopolysaccharides, which alleviated the salt stress on tomato plants. The treated plants were also characterized by higher length and weight of their shoot and root systems, higher K^+^ concentration, and K^+^/Na^+^ ratio [166]. Treating water-stressed whole grains of wheat plants with water extracts of *Chlorella ellipsoida* and *Spirulina maxima* resulted in increased total carotenoid, tocopherol, phenolic, and protein contents [167]. Algal extracts are also effective in activating mechanisms that protect plants from oxidative stress, which is caused by the accumulation of reactive oxygen species (ROS), such as the superoxide anion (O^−^_2_.) and hydrogen peroxide (H_2_O_2_). This response is mainly associated with antioxidant compounds, such as carotenoids, cytokinins, α-tocopherol, and ascorbic acid, as well as the activation of enzymes with antioxidant activity, including peroxidase, superoxide dismutase, glutathione reductase, catalase, and ascorbate peroxidase [14,137,138,147,164,166,168].

## 3. Mechanism of Action of Algal Metabolites

The mechanisms of action of the majority of algal metabolites against agricultural pests are not known, and mechanism studies are usually performed against medically important organisms [169]. However, the available information on potentially biopesticidal algal metabolites is presented below.

### 3.1. Inhibition of Photosynthesis

**Figure 6:**
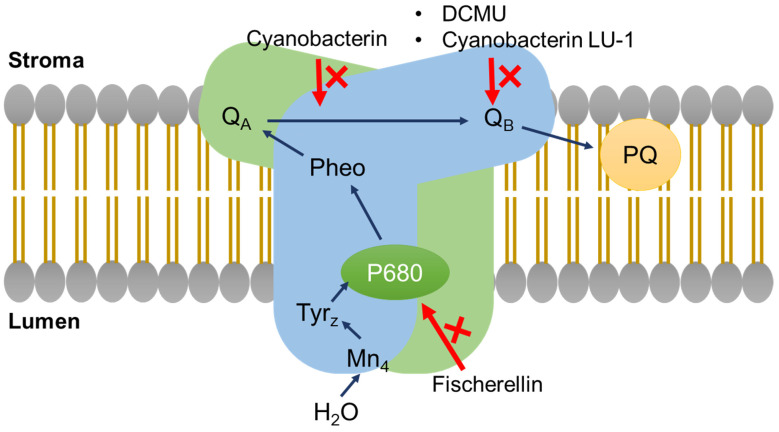
The mode of action of photosynthesis inhibitors as shown on a simplified diagram of photosystem II within the thylakoid membrane. QA = primary quinone acceptor; QB = secondary quinone acceptor; PQ = plastoquinone; Pheo = pheophytin; P680 = photosystem II primary donor; TyrZ = tyrosine-Z radical.

Research on allelopathy between different algal species/genera has led to the discovery of novel secondary metabolites with various modes of action [170]. Among them, photosynthesis inhibitors can potentially be used in higher plants, due to the similarity of photosynthetic apparatus between algae and plants. One of the first photosynthesis inhibitors was isolated from the cyanobacterium *Scytonema hofmanni* UTEX B 2349. Cell extracts or spent medium of this cyanobacterium inhibited the growth of other cyanobacteria and green algae [171]. This led to the isolation of a halogenated secondary metabolite called cyanobacterin (Figure 5) [171]. Cyanobacterin was ineffective against all eukaryotic algae and eubacteria [171,172]. Further work demonstrated thylakoid damage in transmission electron micrographs of *Synechococcus* sp. ATCC 27,146 cells that were exposed to the minimum effective dose of 4.6 µM cyanobacterin. Cyanobacterin also inhibited light-dependent oxygen evolution in the same cyanobacterium, suggesting specific action on photosynthetic electron transport [172]. Cyanobacterin was toxic to aquatic *Lemna* species and terrestrial angiosperms [29]. Similar to *Synechococcus* sp. ATCC 27146, photosynthetic electron transport was inhibited by cyanobacterin in purified pea chloroplasts. Both cyanobacteria and plant studies suggested that the potential site of action of cyanobacterin was a site in PSII (Figure 6) [29]. The site of action proved to be different from the site of action of 3-(3,4-dichlorophenyl)-1,1-dimethylurea (DCMU), which is the quinone binding protein (Q_B_) of PSII [173]. Cyanobacterin did not have any effect on PSI activity [29]. A cyanobacterin-resistant strain of the cyanobacterium *Anacystis nidulans* R2 was selected through mutagenesis with nitrosoguanidine. The mutant was not resistant to 3-(3,4-dichlorophenyl)-1,1-dimethylurea, or DCMU and the authors concluded that cyanobacterin inhibited electron flow from quinone-A to quinone-B [174]. The toxicity of cyanobacterin to non-target organisms was studied in *Daphnia magna*. LC_50_ (48-h) of cyanobacterin in *D. magna* and was approximately 1.37 µg mL^−1^, suggesting caution if this metabolite would ever be used as a herbicide [175].

A secondary metabolite, named cyanobacterin LU-1, that was isolated from *Nostoc linckia* CALU 892, also inhibited the growth of cyanobacteria and algae (Figure 6). This metabolite inhibited the O_2_ evolution in *Synechococcus* sp. PCC 7942 cells in a light-dependent manner. However, its structure and site of action seems to be different compared to cyanobacterin from *Scytonema hofmanni*. Mutants of *Synechococcus* sp. PCC 7942 and *Anabaena variabilis* ALU 458 that were resistant to cyanobacterin LU-1 were also resistant to DCMU, suggesting binding to quinone-B by cyanobacterin LU-1. This metabolite (5 mg) was non-toxic to mice by intraperitoneal injection [127].

The spent culture medium and the cell-free extract of an *Oscillatoria* strain that was isolated from a lake in India inhibited the growth of several cyanobacteria and algae. Ether extract of this strain inhibited oxygenic photosynthesis in *Anacystis nidulans* [176], and the growth of angiosperm plants, presumably through inhibition of electron transport at a site that was different than those of cyanobacterin and DCMU [177]. The cell-free extracts were non-toxic to mice. The active metabolites in ether extract seemed to consist of saturated fatty acids [177].

Nostocarboline (Figure 5) from Nostoc 78-12A is a β-carboline alkaloid inhibiting cholinesterase [178]. Nostocarboline and its derivatives inhibit the growth of cyanobacteria and green algae. The inhibitory activity seemed to be related to the inhibition of photosynthesis in cyanobacteria, since this alkaloid was not active against non-photosynthetic bacteria [129]. Further work proved nostocarboline to be a mammalian serine protease inhibitor. Therefore, photosynthetic inhibition in cyanobacteria and algae by nostocarboline might be related to the inhibition of serine proteases in cyanobacteria and algae [179].

Fischerellin A (Figure 1) was isolated from the cyanobacterium *Fischerella muscicola* UTEX 1829, which inhibited the growth of cyanobacteria and green algae. It did not affect the growth of bacteria. Fischerellin specifically inhibited the photosynthetic electron transport involving PSII in the cyanobacterium *Anabaena* sp. P9 (Figure 1) [180] and in the aquatic macrophyte *Lemna minor* [181]. Later, Srivasatava et al. [182] demonstrated that fischerellin inactivated the reaction centre of PSII and modified the PSII antenna architecture. Apart from its herbicidal effect, fischerellin effectively inhibited the growth of the fungi/oomycetes *Uromyces appendiculatus*, *Erysiphe graminis*, *Phytophthora infestans*, and *Pyricularia oryzae* [181].

### 3.2. Induction of Plant Defense Responses

Exposure to pathogens triggers several defense-related pathways in plants. This is achieved through signal molecules called elicitors that are recognized by receptors on the plant cell membrane [183]. Elicitors may originate from the pathogen or from the plant itself [67,85], or they may be a molecule from a non-pathogenic unrelated organism [183].

**Figure 7:**
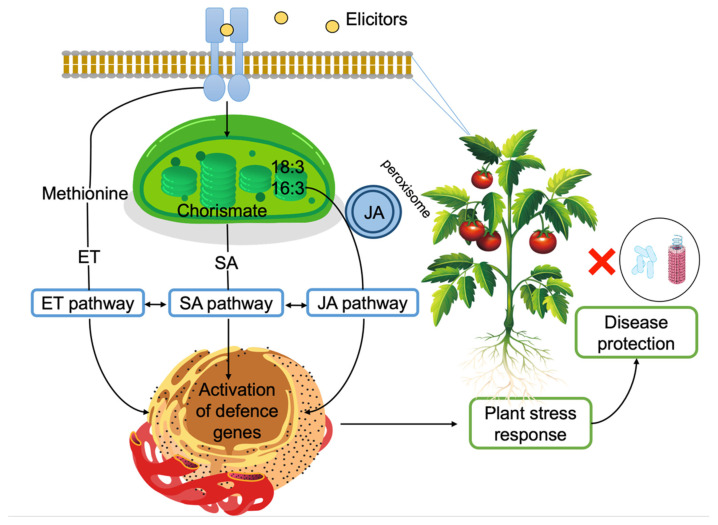
Induction of plant defense responses via elicitors. Elicitors are recognized on the plasma membrane. Jasmonic acid (JA) biosynthesis starts with α-linolenic acid (18:3) or hexadecatrienoic acid (16:3) in the chloroplast. Conversion to JA takes place in the peroxisome [201]. Salicylic acid (SA) biosynthesis starts with chorismate in the chloroplast and SA is transported into the cytosol [202]. Ethylene (ET) biosynthesis starts with methionine and ET is recognized by receptors on the endoplasmic reticulum. All three pathways eventually lead to defense gene expression and evidence suggest crosstalk between these pathways [203].

The principal cell wall polysaccharides from marine macroalgae, ulvans, alginates, fucans, laminarin, and carrageenans, can activate defense mechanisms in plants against pathogens (Figure 7). This response is mainly associated with the generation of oxidative burst and the activation of the salicylic acid (SA), ethylene, and jasmonic acid (JA) signalling pathways [50].

Therefore, this property allows algae and their compounds to be used as plant protectants [184]. One advantage of algal elicitors is that, contrary to pathogens, they don’t usually induce cell death in plants [85]. One notable example is carrageenans (Figure 4) that are found in the cell wall of red algae. Mercier et al. [185] compared the elicitor responses of the pathogen oomycete *Phytophthora parasitica* var. *nicotianae* and, Ƙ-, Ɩ-, and λ-carrageenans in tobacco plants. After infiltration into the mesophyll layer of tobacco leaves, the pathogen caused desiccation and necrosis in the infiltrated area in 24 h. Enzymes that are involved in defense pathways, including sesquiterpene cyclase, chitinase, and proteinase inhibitor type II, were induced, but the activities usually disappeared in 24 h. JA, ethylene, and SA pathways were activated in plants by the pathogen. After the application of λ-carrageenan, macroscopic changes that were similar to the ones that are induced by the pathogen were observed. However, complete necrosis was not recorded. Among the defense genes, sesquiterpene cyclase, chitinase, and proteinase inhibitor type II were induced in plants, and high transcript abundance continued for at least a week for the latter two genes. Similar to the pathogen, JA, ethylene, and SA pathways were activated in plants, albeit for longer durations [185]. The same authors suggested that the perception of algal polysaccharides might be initiated by receptors on the plasma membrane.

Similarly, Sangha et al. [186] investigated the effects of Ɩ- and λ-carrageenans on the induction of defense responses in *Arabidopsis thaliana* ecotype Col-0 against the phytopathogenic fungus *Sclerotinia sclerotiorum*. While λ-carrageenan-treated plants were resistant to infection, the ones that were treated with Ɩ-carrageenan were susceptible. JA pathway-related genes were induced in the former. Additionally, increased oxalase and oxidase activity were detected in plants that were treated with λ-carrageenan, offsetting the oxalic acid that is secreted by the fungus for infection. The authors attributed the different responses of the plant to Ɩ- and λ-carrageenans and to the higher sulfation in the latter [186].

Laminarin, a linear β-1,3 glucan (Figure 4) is a long-term storage compound in brown algae [187]. Incubation of tobacco cv BY cells with laminarin triggered multi-step defense responses, including rapid alkalinization of the culture medium and H_2_O_2_ release, induction of the phenylpropanoid pathway that was demonstrated by a strong Phe ammonia-lyase (PAL) activity at 4 h, caffeic acid O-methyltransferase up-regulation at 2 h until 25 h, and a strong increase in SA from 6 h to 48 h. Similarly, the fatty acid pathway was induced, as demonstrated by lipoxygenase (LOX) activity that was induced within four hours of incubation with laminarin. On the other hand, infiltration of tobacco cv Samsun NN plants with laminarin resulted in the induction of four families of pathogenesis-related (PR) proteins (PR1, PR2, PR3, and PR5), which have antimicrobial activities. All these defense reactions protected the tobacco leaves against infection by the bacterium *Erwinia carotovora* subsp. *carotovora,* which causes soft rot disease [188]. Similarly, laminarin induced defense responses in grapevine (*Vitis vinifera* L.), which included H_2_O_2_ release, calcium uptake, alkalinization of the culture medium, and induction of mitogen-activated protein kinases. The defense responses in the grapevine also included the upregulation of defense-related genes, such as 9-lipoxygenase (*Lox*) and glutathione-S-transferase (*Gst*), phenylalanine ammonia lyase (*Pal*), stilbene synthase 1 (Sts1), polygalacturonase-inhibiting protein (*Pgip*), basic class I, acidic class III, and acidic class IV chitinase genes, class I β-1,3-glucanases gene *Glu1*, and serine protease (*Pin*) genes. Laminarin also caused the production of antimicrobial phytoalexins in the grapevine. These defense reactions protected grapevine against *Botrytis cinerea* and *Plasmopara viticola* infections [85].

The presence of β-1,3-glucans in the unicellular eukaryotic members of Bacillariopyhta and Chryosophyceae, and in *Nannochloropsis gaditana* of Eustigmatophyceae [189,190] opens new avenues of research for their effects in plant defense metabolism. It also provides an opportunity to mass culture these microalgae, preferably within a biorefinery approach, where β-1,3-glucan-separated biomass can be used for other purposes (e.g., feed supplement, pigment source, biofuel feedstock).

An extract of *Ulva* spp. was tested as an elicitor in *Medicago truncatula*. After the application of *Ulva* extract, the expression patterns of mainly defense-related genes were undertaken using a macroarray. In addition to the aforementioned defense-related genes, the upregulation of genes for the cell wall proteins calmodulin, ribonuclease, and aquaporin was observed. Genes that were involved in carbon and nitrogen metabolism were not down-regulated, suggesting that the induction of defense genes did not affect primary metabolism. The *Ulva* extract protected *M. truncatula* against the fungal pathogen *Colletotrichum trifolii*, which causes anthracnose disease [67].

Tobacco leaves that were treated with sulphated fucans (Figure 4) exhibited defensive responses against the tobacco mosaic virus. The oligofucans, in this case, also stimulated the SA pathway and the expression of pathogenesis-related proteins [191].

During a fungal infection, some plant species recognize the chitin (poly-(1→4)-β-linked N-acetyl-D-glucosamine) components in fungal cell walls and induce lignification as a defense mechanism to prevent further fungal damage. This was initially demonstrated in wounded wheat leaves when crab and fungal chitin, chitosan, and ethylene glycol chitin were applied as elicitors [192]. Later work demonstrated the induction of chitinase in melon seedlings upon treatment with varying concentrations of chitin oligosaccharides. The same study suggested the hexamer or heptamer chitin oligomer as the optimal size for maximum chitinase activity [193]. Kaku et al. [194] identified a high-affinity binding protein for chitin in the plasma membrane of rice cells. The knockdown of the receptor gene by RNA interference shut down the chitin-induced defense responses. Further work demonstrated that, in addition to this membrane receptor glycoprotein, a receptor kinase was also required for chitin-based elicitor response in rice [195].

Chitin is naturally found in the exoskeletons of crustaceans and the cell walls of fungi. Industrial production of chitin utilizes crustaceans, such as crabs and shrimp. The isolation of chitin from these sources is a lengthy process, including treatment with HCl and then with NaOH, increasing the cost of production [196]. Less known is the presence of chitin in some algal groups, such as green algae and diatoms [197]. Especially some diatom species from the *Cyclotella* and *Thallassiosira* genera synthesize chitin as nanofibers and extrude through pores on the frustule. The extruded chitin nanofiber is in the form of β-chitin, which is easier to dissolve and modify compared to α-chitin [198]. Moreover, extruded chitin can be readily isolated from the culture medium.

Within a biorefinery approach, a diatom biomass can be produced to obtain various valuable chemicals, including lipids and chitin [199]. The leftover diatom frustules will also have the potential to be used as an insecticide. Diatomaceous earth (DE) is formed from fossilized diatoms dating back 20 to 80 million years. DE contains about 50% moisture and up to 94% silica of dry weight. After mining, the moisture is reduced, and the DE is milled to reduce the particle size to between 10–50 µm. DE is safe for humans, and among many uses, it is also an effective insecticide. After application, DE dust is picked up by insect bodies, where DE damages the wax coat of insects. This results in water loss from the body of insects, causing desiccation and death [200]. Therefore, leftover diatom frustules would be a valuable side product, where all components of diatom biomass can be utilized.

### 3.3. Inhibition of Quorum Sensing

Although algal extracts or metabolites exert antimicrobial properties, knowledge on the mechanism of action of these substrates against plant pathogens is limited. A notable example is the production of halogenated furanones by the red alga *Delisea pulchra*. Early investigations revealed that *D. Pulchra* produced secondary metabolites that prevented fouling on its surface [204]. These secondary metabolites, namely halogenated furanones, were proposed to biomimic acylated homoserine lactones (AHLs) [205], which are signalling molecules that are involved in quorum sensing for the colonization of Gram-negative bacteria [206]. This led Manefield et al. [207] to test a synthetic halogenated furanone for the inhibition of *N*-3-oxohexanoyl-L-homoserine lactone (3-oxo-C6-HSL) in the plant pathogen *Erwinia carotovora* subsp. *carotovora*. Its metabolite 3-oxo-C6-HSL regulated the production of extracellular degradative enzymes and a carbapenem antibiotic, where both processes help the bacterium to infect terrestrial plants [207]. The results suggested that the halogenated furanone inhibited carbapenem production and reduced the production of extracellular enzymes, the exact mechanisms of which were not clear. Other work demonstrated that *D. pulchra* furanones might displace AHL from its binding site, which is a transcriptional activator (i.e., Lux R protein) [208,209]. Follow-up work by Manefield et al. [210] failed to detect furanone binding to the LuxR protein that is expressed in *E. coli*. Instead, their results showed decreased concentrations of cytoplasmic LuxR protein when furanone is added, which led them to suggest halogenated furanones interacted with LuxR causing conformational change for rapid degradation.

Quorum sensing inhibitors were also detected in the microalgae *Chlamydomonas reinhardtii* 2137 [211], *Chlorella saccharophila* CCAP211/48 [212], and the cyanobacterium *Lyngbya majuscula* [213,214]. Therefore, both macro- and microalgae (including cyanobacteria) are unique and largely untapped resources for discovering quorum sensing inhibitors.

### 3.4. Neurotoxicity

Although there is a lack of information regarding the mechanism of action of algal metabolites against agricultural pest insects, limited work that was performed with other insects might provide some clues. Watanabe et al. [215] reported the insecticidal activities of two halogenated monoterpenes, aplysiaterpenoid A and telfairine (Figure 3), from the red alga *Plocamium telfairiae*. Aplysiaterpernoid A and telfairine caused 60% and 80% mortality in the German cockroach (*Blatella germanica*), respectively. Telfairine also caused random firings in the central nervous system, similar to the effects of (1246/35)-1,2,3,4,5,6-hexachlorohexane (γ-BHC), an organochlorine insecticide. Their modelling studies suggested that both aplysiaterpenoid A and telfairine might act on the picrotoxinin receptors of insects.

Extracts of the macroalga *Prasiola crispa* were shown to possess insecticidal properties in fruit fly (*Drosophila melanogaster*) and cockroach (*Nauphoeta cinerea*) [216]. In a follow-up study, *Prasiola crispa* extracts and its phytosterols, namely β-sitosterol, campesterol and stigmasterol (Figure 3), exhibited insecticidal activities in the cockroach *Nauphoeta cinerea* [217]. To understand the mechanism of action of the extracts and isolated compounds, the authors performed acetylcholinesterase activity assays, semi-isolated heart preparations to determine heartbeats, grooming activity assays, and cockroach neuromuscular function assays. The cockroach brain preparations did not reveal any acetylcholinesterase activity. However, the algal extract caused a significant and reversible drop in the heartbeats in the cockroach semi-isolated heart preparations and a significant change in the grooming activity. The extracts and the aforementioned phytosterols also caused neuromuscular inhibition, which was reduced by the application of phentolamine, an octopamine receptor inhibitor. Altogether, these results suggested the involvement of octopaminergic-cholinergic pathways in the insecticidal activity of this Antarctic macroalga [217].

### 3.5. Production of Antimetabolites

An antimetabolite may bind to an enzyme as the native substrate does; however, it is not converted to a functional product. Therefore, antimetabolites may compete with the natural substrates and may block an enzyme’s catalytic activity. Brilisauer et al. [134] observed growth inhibition of the cyanobacterium *Anabaena variabilis* ATCC 29,413 by the spent culture media of the cyanobacterium *Synechococcus elongatus* PCC 7942. The isolation and chemical characterization of the extracellular inhibitory compounds proved the presence of 7-deoxysedoheptulose (7dSh) (Figure 5). Incubation of *A. variabilis* with purified 7dSh resulted in the accumulation of 3-deoxy-D-arabino-heptulosonate 7-phosphate (DAHP) in cells, which is the natural substrate of 3-dehydroquinate (DHQ) synthase. The results suggested that 7dSh was the antimetabolite of DAHP, the native substrate of the DHQ synthase in the shikimate biosynthesis pathway [134,218]. Accordingly, the inhibition of this pathway disturbed protein synthesis by inhibiting aromatic amino acid biosynthesis [134]. The shikimate pathway occurs in bacteria, fungi, and plants, but not in animals [219]. As expected, 7dSh inhibited the growth of *Saccharomyces cerevisiae* and *Arabidopsis thaliana*. No cytotoxicity was detected in mammalian cells, potentially making 7dSh an ideal antibacterial, antifungal, or herbicidal compound.

### 3.6. Blocking Virus Entry into the Plant

Sodium alginate (Figure 4) is a commercial gelling product that is obtained from brown seaweeds [220]. Sano et al. [21] investigated the inhibitory activity of alginate on the infection of tobacco leaves with tobacco mosaic virus (TMV). This polysaccharide did not inactivate TMV. However, its application on leaves reduced TMV infectivity, with the reduction being proportional to the salginate concentration and stiffness of the polysaccharide. The addition of sodium alginate caused TMV particles to form large aggregates on the cell surface, blocking TMV RNA entry into the plant cells. The authors also suggested that anions of the polysaccharide might have interacted with the cationic amino groups of TMV, preventing interaction with the anionic phosphate groups of the cell membrane.

## 4. Conclusions

Extensive research in the past decades on macro- and microalgal species has resulted in the discovery of an impressive array of extracts and bioactive compounds with pesticidal action for application in agricultural practices. These compounds have been used effectively as standalone treatment against agricultural pests, but their full potential can be revealed by combining them with other techniques in the context of integrated pest management. Moreover, even though a lot of progress has been recorded so far in deciphering their mode of action, more knowledge is expected to be produced in the future in this regard. Conservative estimates suggest the number of current algal species to be over 70,000 [221]. Yet, only a small number of these species have been investigated for their effects on other organisms (e.g., antimicrobial effects), mainly in the area of medicine. Concerning the long evolutionary history of algae and their distribution in highly diverse ecological settings from freshwaters to terrestrial ecosystems, one can expect a myriad of different metabolites from algae. This seems to be the case with novel secondary metabolites that are continuously reported from various algal species. Based on the discovery of active metabolites in medicine, where most of the work is done, similar results against plant pathogens can be expected. A limited number of biopesticidal extracts or compounds from algae have usually been reported from seaweeds or cyanobacteria. Therefore, macro- and microalgae represent a large untapped resource for the discovery of novel biopesticide compounds.

## Figures and Tables

**Table 1:** Summary of algal compounds and their antibacterial activities.

Algal Species	Compound/Type of Extract	Target Organism	Disease/Pathogenic Phenotype/Significance	Protected Plant/Organism	Mode of Action	Reference
*Sargassum wightii*	Methanolic extracts	*Pseudomonas syringae*	Leaf spot disease	*Gymnema sylvestre*	NK	[32]
*Gracilaria edulis, Sargassum wightii*, *Enteromorpha flexuosa*	Petroleum ether extracts, methanolic extracts, unsaponified and lipophilic fractions	*Xanthomonas oryzae*	Bacterial blight	Rice plants	NK	[16]
*Sargassum wightii*	Sulphoglycerolipid (methanol extract)	*Xanthomonas oryzae*	Bacterial blight	Rice plants	NK	[33]
*Ulva fasciata*	Methanolic extracts	*Xanthomonas campestris*, *Erwinia carotovora*	Plant pathogens	Various plant species	NK	[34]
*Cystoseira myriophylloides, Fucus spiralis*	Aqueous extracts	*Agrobacterium tumefaciens*	Crown gall disease	*Solanum lycopersicum*	NK	[18]
*Sargassum latifolium*, *Hydroclathrus clathratus, Padina gymnospora*	Methanolic extracts	*Ralstonia solanacearum, Pectobacterium carotovorum*	Brown rot disease	Potato plants	Induction of plant defenses, formation of bioactive secondary metabolites	[35]
*Lessonia trabeculate, Macrocystis integrifolia*	Ethanolic extracts	*Erwinia carotovora Pseudomonas syringae*	Plant pathogens	Various plant species (tomato, *Arabidopsis*, potato plants)	NK	[17]
*Ulva lactuca, Gelidium serrulatum*	Alkaline extracts	*Xanthomonas vesicatoria*	Plant pathogen	Tomato plants (in vitro)	Induction of plant defenses	[49]
*Ulva lactuca, Sargassum filipendula, Gelidium serrulatum*	Alkaline extracts	*Xanthomonas vesicatoria*	Plant pathogen	Tomato plants (in vivo)	Induction of plant defenses	[49]
*Anabaena variabilis, A*. *circinalis*	Ethyl acetate extracts	*Aeromonas* sp.	Skin infections, ulcers, hemorrhagic and septicemic infections	Fish	NK	[48]

NK: not known.

**Table 2:** Summary of the antiviral activities of algal compounds against plant pathogens.

Algal Class/Species	Compound/Type of Extract	Target Organism	Disease/Pathogenic Phenotype	Protected Plant/Organism	Mode of Action	Reference
Phaeophyceae (brown seaweeds)	Sodium alginate	Tobacco mosaic virus (TMV)	Mottling and discoloration on leaves	*Nicotiana tabacum*	Aggregation of viral particles, blocking of decapsulation process	[21]
*Tichocarpus crinitus*	Kappa/beta-carrageenan	Tobacco mosaic virus (TMV)	Mottling and discoloration on leaves	*Nicotiana tabacum*	Plant tissue resistance, effect on the plant genome	[52]
*Tichocarpus crinitus*	Kappa/beta-carrageenan	Potato virus X (PVX)	Crinkle symptoms/plant death	*Datura stramonium*	Stimulation of lytic processes	[22]
*Fucus gardneri, Alaria marginata, Ralfsia sp., Codium fragile, Fragilaria oceanica, Egregia menziesii*	Methanolic extract (alginate)	Potato virus X (PVX)	Crinkle symptoms/plant death	*Chenopodium quinoa*	Aggregation of viral particles	[23]
*Ulva pertusa*	Lectins	Tobacco mosaic virus (TMV)	Mottling and discoloration on leaves	*Nicotiana glutinosa, Chenopodium amaranticolor*	NK	[53,54]
13 species tested – *Cystoseira balearica, Lophocladia lallemandii*, and *Gastroclonium clavatum* exhibited the strongest effect	Lipid extracts	Tobacco mosaic virus (TMV)	Mottling and discoloration on leaves	*Nicotiana tabacum*	NK	[19]
*Durvillaea antarctica*	Aqueous and ethanolic extracts	Tobacco mosaic virus (TMV)	Mottling and discoloration on leaves	*Nicotiana tabacum*	NK	[17]
*Ulva clathrata, Cladosiphon okamuranus*	Sulphated polysaccharides	Newcastle disease virus (NDV)	Respiratory infection, enteric disease, mortality	Poultry	Inhibition of cell–cell fusion	[55]

NK: not known.

**Table 3:** Antifungal activity of algal extracts against known plant pathogens.

Algal Species	Compound/Type of Extract	Target Organism	Disease/Pathogenic Phenotype/Significance	Protected Plant/Organism	Mode of Action	Reference
*Nostoc* sp.	Ethanolic extracts	*Armillaria* sp., *Fusarium oxysporum* f. sp. *melonis*, *Penicillium expansum*, *Phytophthora cambivora*, *P. cinnamomi*, *Rhizoctonia solani*, *Rosellinia*, sp., *Sclerotinia sclerotiorum, Verticillium albo-atrum*	Plant pathogens	In vitro (action against *Sclerotinia sclerotiorum* was verified in the presence of tomato plant).	Induction of plant defenses	[20]
*Cystoseira myriophylloides, Laminaria digitata, Fucus spiralis*	Aqueous extracts	*Verticillium dahliae*	Verticillium wilt disease	Tomato seedlings	Induction of plant defenses	[18]
*Lessonia trabeculata*	Ethanolic extracts	*Botrytis cinerea*	necrotic lesions in leaves	Tomato plants	NK	[17]
*Gracillaria chilensis*	Aqueous and ethanolic extracts	*Phytophthora cinnamomi*	Plant pathogen	In vitro	NK	[17]
*Sargassum latifolium, Padina gymnospora*	Methanolic extracts	*Fusarium solani, Rhizoctonia solani*	Plant pathogens	In vitro, in vivo (*Solanum melongena*)	Induction of plant defenses, formation of bioactive secondary metabolites	[35]
*Ulva lactuca, Sargassum filipendula, Gelidium serrulatum*	Alkaline extracts	*Alternaria solani*	Plant pathogen	Tomato plants	Induction of plant defenses	[49]
*Laminaria digitata, Undaria pinnatifida, Porphyra umbilicalis, Eucheuma denticulatum Gelidium pusillum*	Fatty acids, polysaccharides, phlorotannins	*Botrytis cinerea, Monilinia laxa, Penicillium digitatum*	Postharvest pathogens	In vitro, in vivo (*Fragaria × ananassa*, *Prunus persica*, *Citrus limon*)	Direct toxicity of fatty acids, induction of plant defenses	[68]
10 algal species - *Cystoseira balearica, Codium effusum* and *Codium coralloides* exhibited the strongest effect	Lipid extracts	*Phoma tracheiphila*	Mal secco disease	In vitro	NK	[19]
*Ulva fasciata*	Ulvan	*Fusarium oxysporum* f. sp. *phaseoli*	Bean Fusarium wilt	*Phaseolus vulgaris*	Induction of plant defenses, reduced fungal colonization in plant tissues	[78]
*Ulva fasciata*	Ulvan	*Colletotrichum lindemuthianum*	Anthracnose	*Phaseolus vulgaris*	Induction of plant defenses	[79]
*Ulva armoricana*	Aqueous extracts (ulvan)	*Erysiphe polygoni, E. necator, Sphareotheca fuliginea*	Plant pathogens	*Phaseolus vulgaris,* grapevine plants, *Cucumis sativus*	Induction of plant defenses	[80]
*Ulva fasciata*	Ulvan	*Blumeria graminis*	Plant pathogen	*Triticum aestivum* cv. Kanzler, *Hordeum vulgare* cv. Villa	Induction of plant defenses	[82]
*Ulva fasciata*	Sulphated polysaccharides, alcoholic extracts	*Colletotrichum lindemuthianum*	Anthracnose	In vitro, *Phaseolus vulgaris*	Induction of plant defenses	[83]
*Laminaria digitata*	Laminarin	*Botrytis cinerea, Plasmopara viticola*	Plant pathogens	Grapevine plants	Induction of plant defenses	[85]
*Anabaena* sp., *Ecklonia* sp., *Jania* sp.	Aqueous extracts containing polysaccharides	*Botrytis cinerea*	Grey mold (postharvest plant pathogen)	Strawberry plants	Direct effect, induction of plant defenses	[86]
*Ulva lactuca, Caulerpa sertularioides, Padina gymnospora, Sargassum liebmannii*	Polysaccharide-rich extracts	*Alternaria solani*	Plant pathogen	Tomato plants	Induction of plant defenses (*Ulva lactuca*)	[87]
*Ulva* sp.	Ulvan	*Colletotrichum* *gloeosporioides*	*Glomerella* leaf spot (GLS) disease	Apple plant seedlings (*Malus domestica*)	Induction of plant defenses	[88]
*Ulva fasciata Enteromorpha flexuosa*	Ethyl acetate, benzene, acetone, methanolic and chloroformic extracts	*Macrophomina phaseolina Fusarium solani*	Plant pathogens	Cucumber plants	NK	[89]
*Gracilaria confervoides*	Chloroformic extracts	*Rhizoctonia solani, Fusarium solani, Macrophomina phaseolina*	Plant pathogens	Cucumber plants	NK	[75]
*Sargassum vulgare*	Methanolic extracts	*Pythium aphanidermatum*	Pythium leak disease	Potato plants	NK	[90]
*Sargassum wightii*	Acetone extracts (n-Hexadecanoic acid)	*Rhizoctonia solani*	Rice sheath blight	Rice plant	Induction of plant defenses	[91]

NK: not known.

**Table 4:** Summary of algal products and their pesticidal activity against soil nematodes.

Algal Species	Compound/Type of Extract/Product	Target Organism	Protected Plant/Organism	Mode of Action	Reference
*Jania rubens*	Brominated diterpenes	*Allolobophora caliginosa*	In vitro	ΝΚ	[93]
*Nostoc* sp.	Methanolic extracts	*Caenorhabditis elegans*	In vitro	Induction of plant defenses	[20]
*Spatoglossum variabile, Stokeyia indica, Melanothamnus afaqhusainii*	Dry powders	*Meloidogyne incognita*	Eggplant, watermelon	Direct cytotoxic effect, effect on plant metabolism/resistance to stress	[65]
*Spatoglossum variabile*, *Melanothamnus afaqhusainii*, *Halimeda tuna*	Aqueous and ethanolic extracts	*Meloidogyne javanica*	Sunflower, tomato	Induction of plant defenses	[24]
*Sargassum tenerrimum*, *S. swartzii*, *S. wightii*	Ethanolic extracts (dry powders)	*Meloidogyne javanica*	Okra (*Abelmoschus esculentus)*	ΝΚ	[94]
*Stoechospermum polypodioides*	Methanolic extracts	*Meloidogyne javanica*	In vitro	ΝΚ	[95]
*Ecklonia maxima*	Commercial formulation—Kelpak 66 liquid concentrate (cancelled product)	*Meloidogyne incognita*	Tomato plants (*Lycopersicon esculentum*)	ΝΚ	[96]
*Ascophyllum nodosum, Ecklonia maxima*	Commercial formulations—Kelpak (Kelp Products Ltd., Simon’s Town, South Africa), OSMO^®^ (OSMO^®^ International NV, Diksmuide, Belgium)	*Meloidogyne chitwoodi*, *Meloidogyne hapla*	Tomato plants (*Lycopersicon esculentum*)	Interrupt enzymatic activities of hatching process, alter sensory perception of the roots by the nematodes	[97]
*Ascophyllum nodosum*	Commercial formulation—Algaefol^®^ (Chema Industries, Egypt)	*Radopholus similis, Meloidogyne incognita*, *Belonolaimus longicaudatus*	Citrus, tomato, centipede grass	Cytotoxic effect	[98,99,100,101]

NK: not known.

**Table 5:** Summary of algal products and their activity against insects and mites.

	Insecticidal Activity					
Algal Species	Compound/Type of Extract	Target Organism	Disease/Significance	Protected Plant/Organism	Mode of Action	Reference
*Caulerpa racemosa*	Ethanol and water extracts	*Anopheles stephensi, Aedes aegypti, Culex quinquefasciatus*	Disease vectors	-	Toxic effect (larvicidal)	[25]
*Plocamium cartilagineum*	Mertensene, violacene, and derivatives (dibromomertensene and dihydromertensene)	*Tuta absoluta, Schizaphis graminum*	Crop pests	Tomato plants, cereals	Toxic effect (insecticidal, reduced reproduction)	[104]
*Spirulina platensis, Sargassum vulgar*	Water and ethanol extracts	*Spodoptera littoralis*	Crop pest	Cotton plants, tomato, maize etc.	Toxic effect	[27]
*Caulerpa sertularioides, Laurencia johnstonii, Sargassum horridum*	Ethanol extracts	*Diaphorina citri*	Citrus greening disease	Citrus plants	Toxicity, repellent activity	[109]
*Ulva lactuca*	acetone, ethanol, chloroform, methanol, petroleum ether extracts	*Culex pipiens, Spodoptera littoralis*	Disease vector, crop pest	-	Inhibition of adult emergence and larval growth	[117]
*Caulerpa scalpelliformis*	Chloroform, methanol, hexane extracts	*Dysdercus cingulatus, Spodoptera litura*	Crop pests	Cotton seeds, tomato, maize, vegetables	Repellent activity	[118]
*Padina pavonica*	Chloroform, benzene extracts	*Dysdercus cingulatus*	Crop pest	Cotton, citrus, maize	Toxic effect (nymphicidal, ovicidal)	[115]
*Sargassum tenerrimum*	Chloroform, benzene extracts	*Dysdercus cingulatus*	Crop pest	Cotton, citrus, maize	Toxic effect (nymphicidal, oviposition efficacy)	[114]
*Ulva fasciata, U. lactuca*	Methanol extracts	*Dysdercus cingulatus*	Crop pest	Cotton, citrus, maize	Toxic effect (nymphicidal)	[105]
*Nostoc* sp.	Methanol extracts	*Helicoverpa armigera*	Crop pest	Cotton, tomato, rice etc.	Toxic effect (larvicidal)	[20]
*Sargassum wightii, Padina pavonica*	Chloroform, methanol, water extracts	*Dysdercus cingulatus*	Crop pest	Cotton, citrus, maize	Toxic effect (nymphicidal), effect on biophysical parameters	[106]
*Dictyota linearis, Padina minor*	Ethanol extracts	*Aedes aegypti*	Disease vector	-	Toxic effect (larvicidal)	[107]
*Caulerpa scalpelliformis*	Acetone extract	*Culex pipiens*	Disease vector	-	Toxic effect (larvicidal)	[108]
*Microcystis, Oscillatoria, Nodularia, Nostoc, Anabaena*	Hydrophilic, lipophilic extracts	*Aedes aegypti*	Disease vector	-	Toxic effect	[110]
*Ulva lactuca*	Acetone extract	*Drosophila melanogaster*	Fruit fly, model organism	-	Toxic effect	[111]
*Chara vulgaris, Parachlorella kessleri, Ulva intestinalis, Cladophora glomerata, Nostoc carneum*	Ethanol extracts	*Spodoptera littoralis*	Crop pest	Cotton, tomato, maize, vegetables	Toxic effect (larvicidal), effect on biophysical parameters	[113]
		**Acaricidal activity**				
*Ascophyllum nodosum*	Commercial formulation—Maxicrop^®^ (Maxicrop International Ltd.)	*Tetranychus urticae*	Mottled leaves, early leaf loss	Strawberry plant	-	[26]
*Oscillatoria* sp., *Phormidium* sp., *Spirulina platensis, Spirulina maxima, Ulva intestinalis, Sargassum* sp., *Dictyota* sp.	Methanol, dichloromethane, hexane extracts	*Dermatophagoides pteronyssinus*	Disease vector	-	Toxic effect	[112]

**Table 6:** Herbicidal activity of algal compounds.

Algal Species	Compound/Type of Extract	Target Organism	Disease/Significance	Mode of Action	Reference
*Synechocystis aquatilis*	Norharmane	*Microcystis aeruginosa*, *Oscillatoria limnetica*, *Chlorella vulgaris*, *Ulothrix* sp.	Management of algal blooms	Effect on metabolism, effect on the photosynthetic apparatus	[126]
*Synechocystis aquatilis, Nodularia harveyana*	Norharmane	*Avena fatua*, *Plantago lanceolata*, *Portulaca oleracea*, *Echinochloa crusgalli*, *Amaranthus retroflexus*	Crop weeds	Effect on metabolism, effect on the photosynthetic apparatus	[28]
*Schytonema hofmanni*	Cyanobacterin	*Lemna gibba, Setaria viridis, Avena fatua, Rumex crispus, Polygonium convolvulus, Zea mays, Pisum sativum*	Management of phototrophic organisms	Inhibition of photosynthesis	[29]
*Nostoc linckia*, *Schytonema hofmanni*	Cyanobacterins	*Synechococcus* sp.	Management of algal blooms	Inhibition of photosynthesis	[127,128]
*Nostoc* sp., *N. spongiaeforme*	Nostocyclamide, nostocine A, nostocarboline	*Microcystis aeruginosa, Synechococcus* sp., *Kirchneriella contorta, Chlamydomonas reinhardtii, Chorella pyrenoidosa, Chlorella fusca, Dunaliella tertiolecta, D. salina*	Management of algal blooms	Inhibition of photosynthesis, generation of reactiveoxygen species (ROS)	[129,130,131]
*Microcystis aeruginosa*	Microcystins	*Myriophyllum variifolium*, *Lemna japonica*	Management of eutrophic waters	Inhibition of protein phosphatases, cell regulation	[132,133]
*Synechococcus elongatus*	7-Deoxy-sedoheptulose (methanolic extract)	*Anabaena variabilis*, *Arabidopsis*	Management of phototrophic organisms	Inhibition of the shikimate pathway, cell metabolism	[134]
*Nostoc* sp.	Methanolic extract	grass seedlings	Crop weeds	Toxicity, antimitotic agents with inhibitory effects	[20]
